# Corrosion in Modular Revision Hip Stem Tapers – A Retrieval Analysis

**DOI:** 10.1002/jor.70078

**Published:** 2025-10-02

**Authors:** Therese Bormann, Haolan Yan, Sebastian Jaeger, Mareike Schonhoff, J. Philippe Kretzer

**Affiliations:** ^1^ Research Center of Biomechanics and Implant Technology, Department of Orthopaedics Heidelberg University Hospital Germany

**Keywords:** corrosion wear, fretting wear, fretting‐corrosion, revision surgery, taper failure, total hip arthroplasty

## Abstract

In revision hip arthroplasty, modular stems enable intraoperative adjustment of the biomechanics of the hip to ensure a stable joint function even in complex anatomical cases. Modular stem junctions, however, carry the risk of junction degradation due to corrosive processes or even junction breakage. Fretting‐corrosion has been mentioned as precursor of junction breakage but has hardly been systematically assessed. To investigate relations between corrosion and fretting, and implant and patient specific parameters and connection strength, respectively, a collection of 53 retrieved modular hip stems of different implant systems was investigated. Corrosion and fretting at the stem‐neck taper connection were rated with a modified Goldberg score. Taper contamination was assessed with a similar scoring system. If the hip stems were still joint to the neck piece, the push‐out force to detach the parts was recorded as a measure for taper junction strength. A multivariate regression analysis revealed that corrosion and fretting were significantly affected by implantation time, taper contamination, body weight and implant system. Taper junction strength was not altered by corrosion or fretting but by taper contamination. The results indicate that implant geometry parameters are not related to the extent of corrosive degradation at the junction, but taper contamination significantly increased corrosion at taper surfaces. This underlines the importance of the cleanliness of the taper surfaces during hip stem assembly for a long‐term stability of the modular implant.

## 1 Introduction

Modularity in total joint arthroplasty enables intraoperative tailoring of geometric implant parameters to the patient specific situation and partial replacements in case of a revision surgery. Nevertheless, any additional interface in a load bearing implant holds the risk of junction degradation or loosening. Junction degradation caused by mechanically assisted crevice corrosion has been investigated extensively for head‐neck taper connections and also for dual‐neck hip stems [1, 2, 3, 4, 5]. In dual‐neck implants, an increased rate of neck fractures initiated by tribo‐corrosion at the interface between stem and neck was observed [6, 7]. Especially patients with high BMI and implants with a long modular head were shown to be at risk of this kind of failure [7]. Furthermore, excessive fretting‐corrosion at the junction between stem and neck, led to a high incidence of metal‐related adverse local tissue reactions (ALTR) like pseudotumor formation or bone necrosis [2, 8, 9]. A direct comparison of the survivorship of dual‐taper and single‐taper primary hip stems further showed a decreased survivorship of the dual‐taper stems, even with a low incidence for taper breakage or ALTRs [10]. For these reasons, dual‐neck primary hip implants disappeared widely from the market [9]. In revision surgery, however, modularity is still an important feature, as complex revision cases often come along with large bone or soft tissue defects, which make a stable reconstruction of the joint difficult. Such defects can also occur during the course of operation, which makes it impossible to account for all of them already in the planning of the surgery. Corrosion at junction interfaces has raised little attention in modular revision implants, but the problem of junction degradation due to corrosive processes also exists here [11, 12, 13]. Metal‐debris related clinical implications have been for example reported upon taper corrosion in revision total knee arthroplasty [14, 15]. In addition to taper corrosion, modular revision hip stems (MRHS) carry the risk of breakage of the taper connection between hip stem and modular neck piece [16, 17, 18]. The actual rate of taper junction breakage has been controversially discussed [19]. Reported fracture rates range from 0.3% to 4.9%, depending on the investigated data pool [17, 18, 20, 21, 22]. Even though taper junction breakage is a rare complication, the consequences for the patients are fatal with a revision being inevitable [19]. Whether taper breakage is a purely mechanical problem or if corrosion plays a role in the failure has been discussed, but to date there has been no clear consensus on this question [21, 23, 24, 25]. Known risk factors for junction breakage in MRHS are ‐ from the patients side ‐ high body weight, high activity and missing proximal bone support [26, 27] and ‐ from the implant side ‐ short neck pieces and high offsets [20, 21]. Also, corrosion‐fatigue has been described as failure mechanism at taper junctions [24]. In a former investigation, we showed that taper corrosion occurs frequently in clinically inconspicuous taper connections and that the extent of junction degradation was not correlated to geometric implant parameters in a relatively small collection of one MRHS system [11]. Whether these findings apply to larger retrieval collections that comprise different implant systems is an open question. Furthermore, retrieval collections generally comprise the difficulty of differing preconditions of the implants. Therefore, large retrieval series are necessary to recognize influences of patient‐ or implant‐specific factors on implant deterioration. In this study, a collection of 53 retrieved MRHS of several manufacturers was analysed to answer the following questions. (i) To what extent do corrosion and fretting occur in taper junctions of modular revision hip stems? (ii) Is the extent of corrosion and fretting linked to taper contamination, implant manufacturer, or a periprosthetic infection? (iii) Is the extent of corrosion and fretting linked to parameters that are associated with taper breakage? (iv) Is the connection strength altered by corrosive junction degradation?

## 2 Materials and Methods

### 2.1 Retrieval Collection

**Figure 1:**
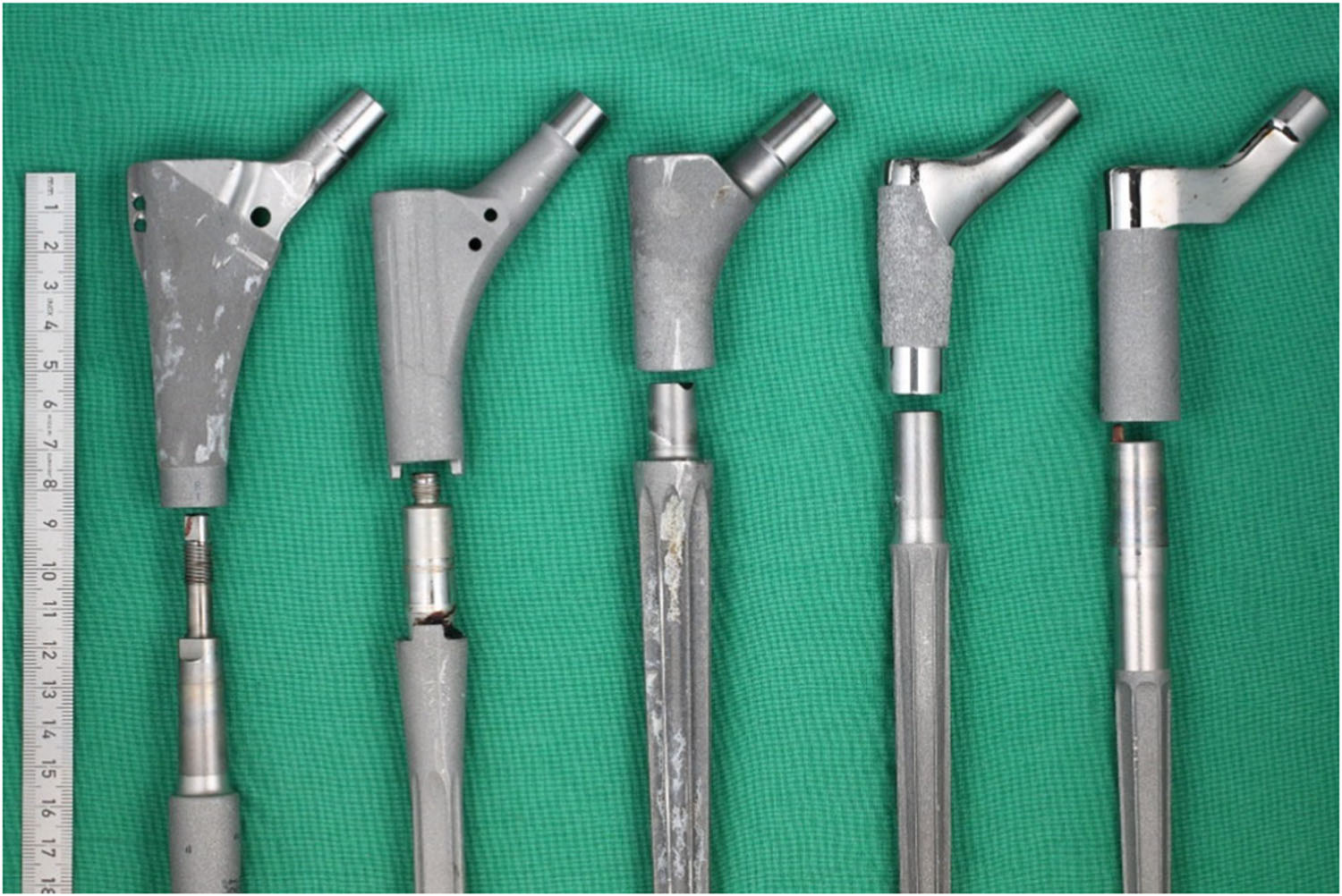
Examples of different types of the investigated modular revision hip stem taper connections. From left to right: Prevision (B. Braun Melsungen AG), PFM‐R (Zimmer‐Biomet), MRP‐Titan (Peter Brehm), Restoration Modular System (Stryker), Reclaim (DePuy Synthes).

The IRB approved retrieval registry of the Department of Orthopaedics at the Heidelberg University Hospital was screened for MRHS. Inclusion criteria were: Collection of the retrieval between 2014 (when retrieval collection was started) and 2022; patient consent to destructive testing; stem and neck piece joint with a taper connection. 64 retrievals met these criteria. Exclusion criteria were fractured modular stems (*n* = 4), large (taper diameter > 16 mm) tapers (*n* = 1) and more than one taper connection in the stem (*n* = 4), which left 55 retrievals. Examples of the different stem types present in the collection are displayed in Figure 1. In the collection, 23 retrievals had still assembled taper connections. In these cases, the neck piece was separated from the stem while measuring the disassembly‐force [11]. In two cases, the parts could not be disassembled. This left 53 retrievals for the investigations. Reasons for revision in this collection were infection (*n* = 23), septic loosening (*n* = 9), aseptic loosening (*n* = 11), luxation (*n* = 6) and periprosthetic fracture (*n* = 4). The demographic data of the retrieval collection is displayed in Table 1. The retrieval collection was further subdivided in four groups with respect to the implant system (Table 2).

**Table 1:** Demographics of the investigated retrieval collection.

Retrieval collection	
*n*	53
Neck piece only	8
Time in situ/years (median/range)	1.8/0.1–15.1
Sex (female/male)	25/28
Body weight/kg (mean ± SD)	80 ± 21
Patient age at implantation/years (median/range)	70/33–85

**Table 2:** Implant systems present in the retrieval collection. If less than five retrievals of one system were present, they were combined in the mixed group.

Manufacturers/Systems	*n*	Group
MRP‐Titan, Peter Brehm GmbH, Weisendorf, Germany	29	MRP‐Titan
Prevision, B. Braun Melsungen AG, Tuttlingen, Germany	5	Prevision
Revitan or PFM‐R, Zimmer‐Biomet Inc., Warsaw, Indiana, USA	12	Revitan/PFM‐R
Reclaim, DePuy Synthes, Raynham, Massachusetts, USA	2	Mixed
Restoration Modular System, Stryker, Kalamazoo, Michigan, USA	2	Mixed
Helios, Biomet Merck, Berlin, Germany	2	Mixed
unknown	1	Mixed

### 2.2 Damage Scoring

The taper connections were rated with respect to corrosion and fretting with a modified Goldberg score [28]. Taper contamination was rated similarly. Internal and external taper surfaces were rated. For all three categories, the damage score scale ranged from 0 to 3. The criteria for damage score assignation are displayed in Table 3. Corrosion products were identified visually by a tarnish with a dull appearance on the surface, which has a colour range from bright (greyish to reddish) to black. The surfaces were scored in eight individual quadrants (proximal‐medial, proximal‐anterior, proximal‐lateral, proximal‐posterior, distal‐medial, distal‐anterior, distal‐lateral and distal‐posterior). If taper engagement length was < 12 mm (Revitan/PFM‐R‐group) the subdivision in distal/proximal zones was dispensed. Rating was done by two individual observers. If the damage score difference between both observers was > 1, the damage score was determined by consensus. For data analysis, the damage score data set of observer 1 was used. Eventually, for each retrieval and damage category the mean value was derived from all individual scores of internal and external taper surfaces, leading to graded scores with a minimum score of 0 and a theoretical maximum score of 3.

**Table 3:** Criteria for assignation of the respective damage score.

Damage score	Corrosion	Fretting	Contamination
0	No corrosion observed	No fretting observed	No or punctiform contamination
1	< 33% of investigated are discoloured	Few fretting marks, located at the distal edge of the taper connection only	Mild contamination without incrustation
2	> 33% of investigated area discoloured or < 10% of area covered by corrosion products	> 10% of investigated shows fretting marks	Contamination with partial incrustation
3	> 10% of investigated area covered by corrosion products	> 50% of investigated area shows fretting marks	Contamination with expanded incrustation

### 2.3 Data Analyses

Statistical analysis was done with IBM SPSS statistics (27.0, IBM Corp., Armonk, NY, USA). Data is presented as mean and standard deviation in case of normally distributed data according to the Shapiro−Wilk test, otherwise, median and range are presented. As corrosion and fretting are correlated, the effect of patient and implant specific parameters on taper corrosion and fretting was investigated by a multivariate multiple linear regression analysis. Investigated independent variables were implantation time, body weight, implant offset, neck piece length, taper contamination, septic/aseptic revision, and implant manufacturer. *Implant offset* took the offset of the neckpiece and the length of the femoral head into account. The data sufficiently met the assumption of multivariate normality, but data for fretting was slightly skewed and had a negative kurtosis. With respect to the variable *manufacturer*, post hoc testing was done using the Games‐Howell test.

The correlation between implantation time and connection strength was assessed with the Pearson correlation coefficient (r). The correlation between corrosion and fretting, and between corrosion, fretting and taper contamination, respectively, with taper connection strength was assessed with Spearman rank's order correlation coefficient (ρ). Level of statistical significance was set to *p* < 0.05.

## 3 Results

### 3.1 Damage Scoring

**Figure 2:**
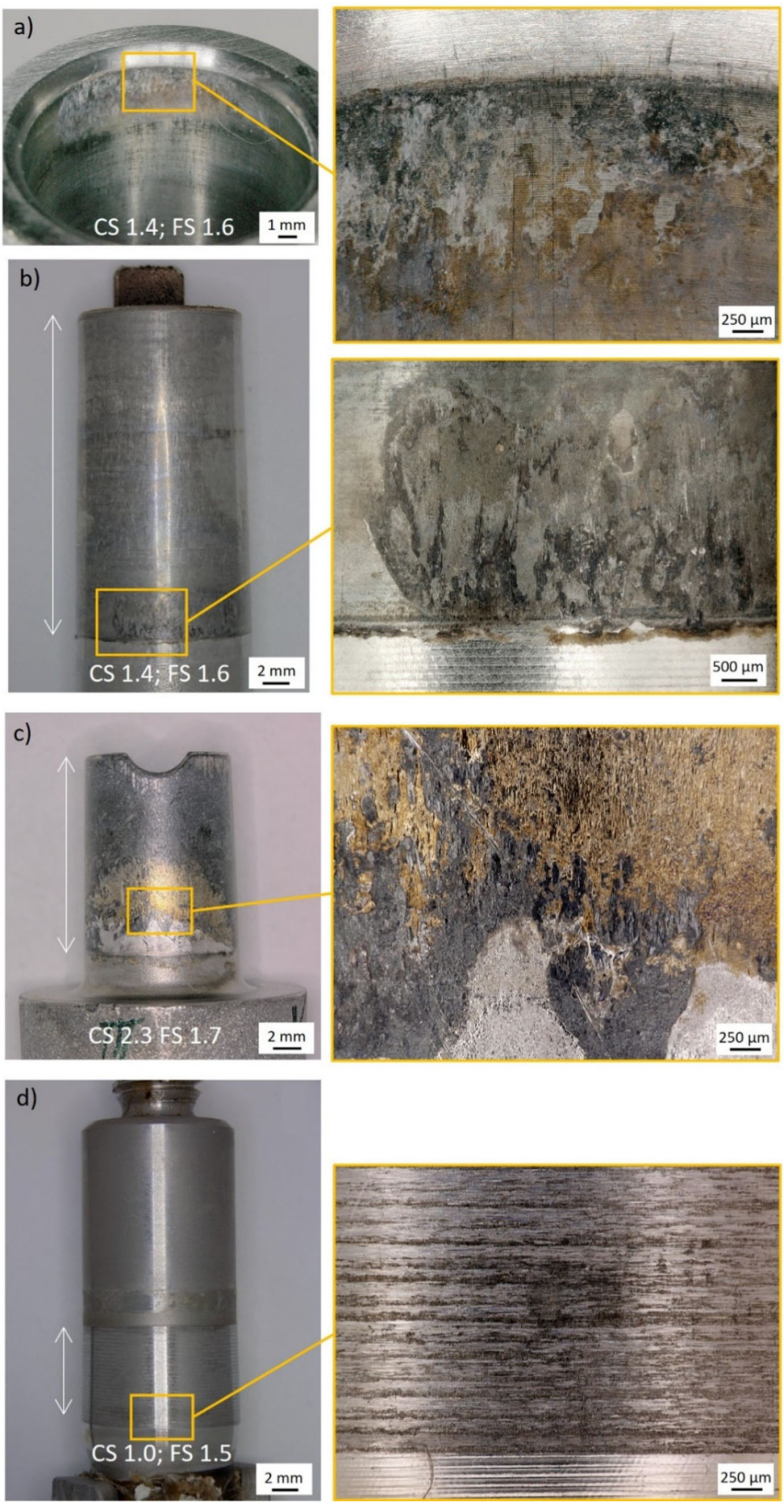
Macro‐ and microscopic images of taper surfaces of different implant systems. Mean corrosion (CS) and fretting scores (FS) of the respective implant are indicated in the image. The white arrow indicates the taper engagement region. (a) and (b) refer to the same implant (Reclaim, DePuy Synthes) after 2 years in situ, (c) MRP‐Titan‐stem after 16.5 years in situ and (d) Revitan/PFM‐R‐stem after 1.5 years in situ.

The investigated collection exhibited a mean corrosion damage score of 1.0 (range, 0–2.4). Median fretting score was 0.8 (range, 0.1–2.1) and median taper contamination score was 0.8 (range, 0–2.6). If the mean damage score for corrosion/fretting was 0.5 or below, corrosion/fretting were considered negligible. This left 35/53 implants with considerable corrosion and 36/53 cases with considerable fretting (see Figure 2). Mean damage scores of more than 0.5 to 1.0 were considered as mild corrosion/fretting and were found in 13 and 18 cases, respectively. Mean damage scores of more than 1.0 to 1.5 were considered as moderate damage and were observed in 12 cases for corrosion and in four cases for fretting. Mean damage scores of > 1.5 were considered as severe damage and were observed in 14 cases for corrosion and in 15 cases for fretting. Corrosion and fretting scores were correlated (ρ = 0.683, *p* < 0.001).

**Figure 3:**
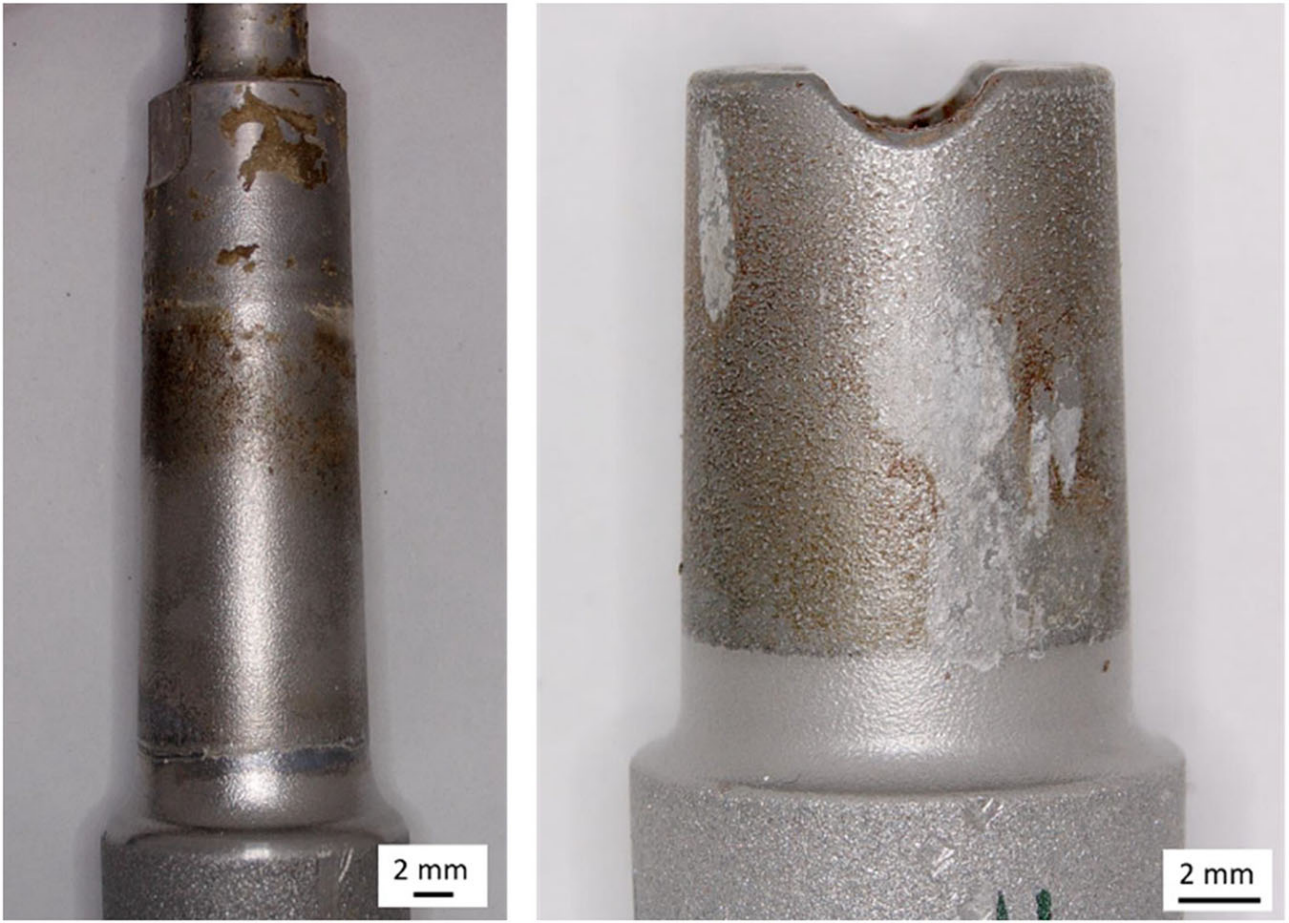
Macroscopic images of taper trunnions exhibiting severe taper contamination (left: mean contamination score 1.6, right: mean contamination score of 2.1).

**Figure 4:**
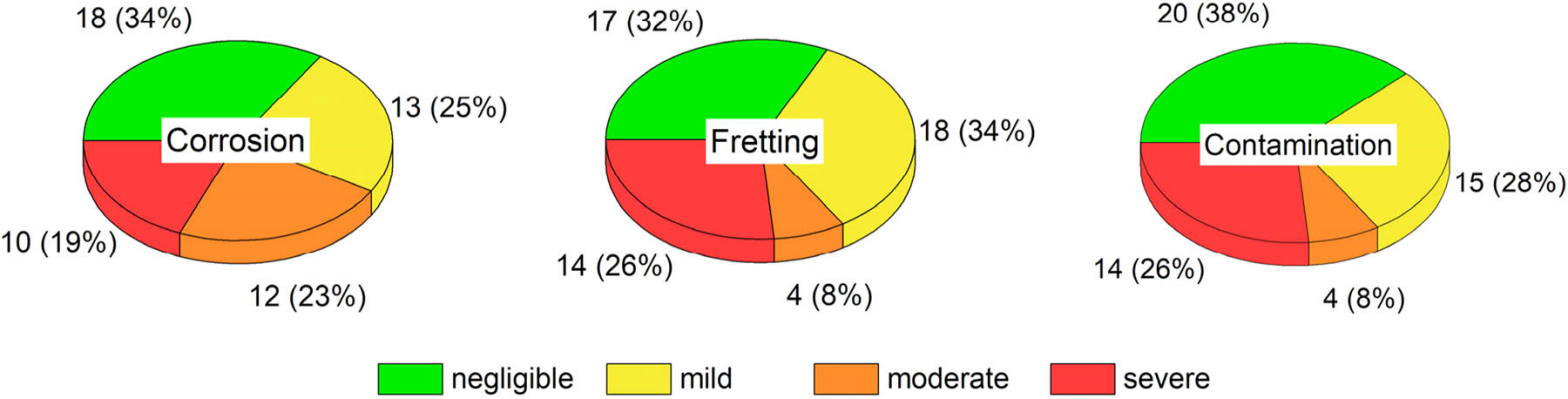
Distribution of corrosion, fretting and contamination damage scores at the taper surfaces in the investigated retrieval collection.

Taper contamination (mean contamination score > 0) was observed in 42/53 cases. Mild contamination was observed in 15, moderate contamination in four and severe contamination (see Figure 3) in 14 cases. Distributions of damage severity are shown in Figure 4.

### 3.2 Factors Associated to the Extent of Corrosion and Fretting

The multivariate regression analysis revealed that corrosion and fretting were significantly influenced by implantation time, taper contamination, body weight and manufacturer, while offset, neck piece length and infection were not correlated to corrosion and fretting. The values for Wilks–Lambda, along with the corresponding F‐ and *p*‐values and partial eta‐squared values of all investigated variables are displayed in Table 4. The test of between‐subject effects revealed a significant effect of time in situ, body weight and taper contamination on corrosion, while body weight and manufacturer had a significant effect on fretting. The corresponding F‐ and *p*‐values as well as the partial eta‐squared values are displayed in Table 5. Post‐hoc testing with respect to manufacturer revealed that for corrosion the group Revitan/PFM‐R differed significantly from all other groups, while for fretting the group Revitan/PFM‐R differed from the groups Prevision and MRP‐Titan.

**Table 4:** Results of the multivariate regression analysis.

Independent variable	Value	*F*	*p*	Partial eta‐equare
Time in situ	0.710	7.946	0.001	0.290
Body weight	0.735	7.044	0.002	0.265
Offset	0.909	1.943	0.157	0.091
Neck piece length	0.947	1.102	0.342	0.053
Taper contamination	0.739	6.881	0.003	0.261
Infection	1.0	0.005	0.995	0
Manufacturer	0.709	2.437	0.033	0.158

**Table 5:** Results of the in‐between subject test for the variables with a significant effect on corrosion and fretting.

Independent variable	Dependent variable	F	*p*	Partial eta‐square
Time in situ	Corrosion	16.281	< 0.001	0.289
	Fretting	3.573	0.066	0.082
Body weight	Corrosion	10.500	0.002	0.208
	Fretting	10.284	0.003	0.205
Taper contamination	Corrosion	13.035	0.001	0.246
	Fretting	0.421	0.520	0.010
Manufacturer	Corrosion	2.529	0.071	0.159
	Fretting	4.569	0.008	0.255

### 3.3 Connection Strength

**Figure 5:**
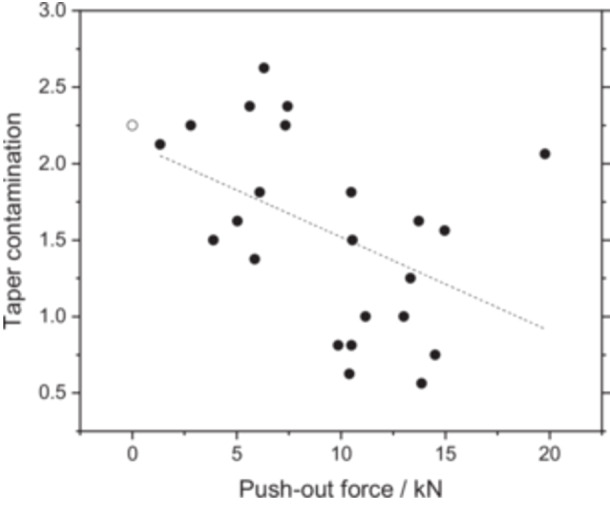
The push‐out force of the modular connections decreases with taper contamination. The blank dot represents one loose connection.

The mean disassembly force of the taper connections was 9.1 kN ± 4.8 kN. There was no correlation between the connection strength and implantation time (r = −0.118, *p* = 0.6), corrosion damage score (ρ = −0.104, *p* = 0.6) and fretting damage score (ρ = −0.137, *p* = 0.5), respectively. Taper contamination, on the other hand, was negatively correlated with the push‐out force (ρ = −0.566, *p* = 0.006), as shown in Figure 5. In one case the assembled parts were loose (push‐put force denoted as 0 kN).

## 4 Discussion

In this study, we investigated fretting and corrosion at the taper junction between stem and neck piece in retrieved MRHS. Evidence of corrosion and/or fretting was found in almost all investigated junctions. However, we considered mean damage scores ≤ 0.5 as negligible, leaving about two third of the collection with considerable corrosion and fretting, respectively. Severe corrosion and fretting were found in 19% and 26% of the cases, respectively. Other studies on retrieval series reported similar values for the occurrence of corrosion and/or fretting in modular stem junctions of hip or knee implants: Kurtz et al. found about 20% of cases exhibiting severe taper corrosion in a collection of 50 modular knee‐implants [13]. Kop et al. investigated a collection of 57 retrieved modular hip stems and found severe corrosion in about 16% of the cases [12]. Accordingly, corrosion and fretting seem to be a general accompaniment of modular junctions in joint arthroplasty, and, therefore, may contribute to metal related clinical complications.

To answer the question, if specific parameters influence the extent of corrosive degradation at this taper junction, a multivariate multiple linear regression analysis was accomplished. The analysis revealed that aside from implantation time, which is known to be related to corrosive junction degradation [29], a significant impact was found for contamination of the taper surfaces, patient weight and implant system. Patient weight was the only investigated variable that influenced fretting and corrosion similarly. Time in situ and taper contamination were related to taper corrosion only ‐ fretting was not affected by these variables. This is somewhat surprising, since fretting and corrosion are closely correlated [28], and fretting is considered as precursor of corrosion. It is, however, possible that initial signs of fretting disappear with increasing corrosive junction degradation. Despite the extensive research on factors that influence taper corrosion at the head‐neck taper junction and the fact that avoiding taper contamination to increase taper stability can be considered as state‐of‐the‐art [30, 31], little evidence of a possible correlation between contamination and taper corrosion exists. We formerly investigated corrosion in modular stem junctions in a different and smaller retrieval collection and found a correlation between increased corrosion and internally polluted neck pieces [11]. The present examination confirms the relation between taper surface contamination and taper corrosion in a larger retrieval collection. Taper contamination can increase micro‐motion at the interface of taper connections [32] that induce damage of the passive layer of the alloys, which, in turn, facilitates corrosive processes [33]. Also, residuals from contamination may act as three‐body in the interface, leading to possible passive layer damage or maybe even chemical alterations within the interface [34].

Manufacturer impacted the extent of fretting, while no significant impact on corrosion was found. The Revitan/PFM‐R‐group showed higher fretting scores than the groups MRP‐Titan and Prevision, while there was no difference between the Revitan/PFR‐M‐group and the mixed group. The taper junction of the Revitan/PFM‐R‐group differs in several ways from the majority of the other investigated tapers. First, the engagement length of the taper junction of the Revitan/PFM‐R‐group is approximately half the length of the taper junctions of most of the other systems investigated, which may lead to an altered response to micromotion upon taper loading. Second, the Revitan/PFM‐R stem taper has a machined surface, while the surfaces of the stem tapers in the groups Prevision and MRP‐Titan are shot peened. The machined surface may be more susceptible to fretting than the generally rougher peened surfaces. Lastly, the Revitan/PFM‐R‐taper connection applies two different metals, a stem taper of CoCr and a neck piece of titanium‐alloy. In all other investigated retrievals, the taper connection was made from titanium‐alloy only. It seems, therefore, that the appearance of fretting is determined by junction specific parameters like taper geometry, surface roughness or material pairing. From head‐neck taper junctions it is known that the combination of mixed metals can lead to increased fretting‐corrosion [28, 35], even though this issue has been controversially discussed [36, 37]. It is interesting to note that in the head‐stem taper connection, the head tapers from CoCr‐alloy wear more than the stem tapers from titanium‐alloy [28, 38]. This behaviour could not be observed in the investigated taper connections with mixed metal combination, here the fretting and corrosion scores did not differ between titanium neck taper and CoCr stem taper.

The geometric parameters implant offset and neck piece length were correlated to an increased risk of taper breakage, as well as a high body weight [21, 26]. Interestingly, body weight impacted corrosion and fretting, while implant offset and neck piece length could not be associated to the extent of junction degradation. Aside from these parameters, corrosion and fretting are generally discussed as precursor or initiation for taper failure [21, 23, 24]. The results of this investigation, however, do not clearly indicate whether corrosive junction degradation is a general accompaniment of increased taper loading, which may eventually result in taper breakage. Fretting‐corrosion at the taper connection appears to be a phenomenon that occurs rather independently from the loading scenario in the interface.

In addition to increasing corrosive processes, taper contamination also affected the push‐out force of the junctions, showing that the connection strength can be negatively impacted if taper surfaces are not properly cleaned before joining the parts. That contamination can influence the connection strength of taper connections has been shown in an in‐vitro study before [30]. It is, however, an interesting observation that taper contamination still leads to decreased push‐out forces even after the implant has been in use for several years. Surface contamination possibly prevents tightening of the connection during activities that impose load on the connection. Interestingly, a lower connection strength might also lead to increasing micromotion at the interface and to associated corrosive processes, but there was no correlation between push‐out force and taper corrosion or fretting. Forces acting in the hip joint during normal activities like walking, stair climbing or standing are in the range of 2–3 times of the body weight, but can raise up to about 8 times of the body weight or more in unforeseen events like stumbling [39]. Such events lead to high bending stresses in the taper connection [40] and may have the potential to induce loosening of the connection. Therefore, aside from taper cleaning, proper securing of the connection with the designated elements is essential to ensure a long lifespan of the connection and thus the implant.

The study has several limitations. First of all, it is a retrieval study, which means that the investigated implants may not represent the majority of well‐working and still‐implanted devices. None of the investigated retrievals was revised directly due to problems with the modular junction, which means that the investigated modular junctions can be considered as representative for MRHS. Nevertheless, it cannot be ruled out that some of the implants were revised because of metal wear related clinical problems, which may hide behind reasons for revision like aseptic stem loosening or mechanical complications. With respect to taper strength of the retrievals, it is unknown, if the taper junctions were already subjected to forces during explantation. It is imaginable that pushing out the stem may have already contributed to loosening of the taper connection. A further limitation is the usage of a modified Goldberg score to assess corrosion and fretting. The scoring method is a semi‐quantitative visual approach, whose ability to correctly represent the extent of taper damage has been debated [36, 41]. However, in retrieval analysis the quantitative determination of taper degradation is often simply impossible, as no unaffected reference state of the samples is present. Furthermore, different modular hip stem systems with varying taper designs were compared. Also, in some cases only neck pieces were present. To overcome the problem of different taper surfaces, the mean value of all individually rated areas was used instead of the sum.

## 5 Clinical Significance

The occurrence of fretting corrosion at taper surfaces, which may be a source for taper failure, can not entirely be precluded when it comes to taper connections in total hip arthroplasty. Therefore, modular revision hip stems should be used precautious and rather in cases where treatment with a monoblock stem has major disadvantages. If modular stems are used, clean taper surfaces should be ensured before connecting stem and neck piece. Manufacturers should include tools that enable cleaning of taper surfaces in the operative environment after the implantation of the stem.

## Author Contributions

**Therese Bormann:** conceptualization, validation, formal analysis, investigation, writing (original draft), project administration. **Haolan Yan:** investigation, formal analysis, writing (review and editing). **Sebastian Jaeger:** cconceptualization, project administration, writing (review and editing); **Mareike Schonhoff:** validation, writing (review and editing). **J. Philippe Kretzer:** methodology, resources, writing (review and editing), supervision.
